# Comparing the Usability and Acceptability of Wearable Sensors Among Older Irish Adults in a Real-World Context: Observational Study

**DOI:** 10.2196/15704

**Published:** 2020-04-20

**Authors:** Alison Keogh, Jonas F Dorn, Lorcan Walsh, Francesc Calvo, Brian Caulfield

**Affiliations:** 1 Insight Centre for Data Analytics UCD School of Public Health, Physiotherapy and Sports Science University College Dublin Dublin Ireland; 2 Data and Digital Novartis Pharma AG Basel Switzerland; 3 Data Sciences Novartis Business Services Dublin Ireland

**Keywords:** wearable technology, usability, mixed methods, user satisfaction

## Abstract

**Background:**

Wearable devices are valuable assessment tools for patient outcomes in contexts such as clinical trials. To be successfully deployed, however, participants must be willing to wear them. Another concern is that usability studies are rarely published, often fail to test devices beyond 24 hours, and need to be repeated frequently to ensure that contemporary devices are assessed.

**Objective:**

This study aimed to compare multiple wearable sensors in a real-world context to establish their usability within an older adult (>50 years) population.

**Methods:**

Eight older adults wore seven devices for a minimum of 1 week each: Actigraph GT9x, Actibelt, Actiwatch, Biovotion, Hexoskin, Mc10 Biostamp_RC, and Wavelet. Usability was established through mixed methods using semistructured interviews and three questionnaires, namely, the Intrinsic Motivation Inventory (IMI), the System Usability Scale (SUS), and an acceptability questionnaire. Quantitative data were reported descriptively and qualitative data were analyzed using deductive content analysis. Data were then integrated using triangulation.

**Results:**

Results demonstrated that no device was considered optimal as all scored below average in the SUS (median, IQR; min-max=57.5, 12.5; 47.5-63.8). Hexoskin was the lowest scored device based on the IMI (3.6; 3.4-4.5), while Biovotion, Actibelt, and Mc10 Biostamp_RC achieved the highest median results on the acceptability questionnaire (3.6 on a 6-point Likert scale). Qualitatively, participants were willing to accept less comfort, less device discretion, and high charging burdens if the devices were perceived as useful, namely through the provision of feedback for the user. Participants agreed that the *purpose of use* is a key enabler for long-term compliance. These views were particularly noted by those not currently wearing an activity-tracking device. Participants believed that wrist-worn sensors were the most versatile and easy to use, and therefore, the most suitable for long-term use. In particular, Actiwatch and Wavelet stood out for their comfort. The convergence of quantitative and qualitative data was demonstrated in the study.

**Conclusions:**

Based on the results, the following context-specific recommendations can be made: (1) researchers should consider their device selection in relation to both individual and environmental factors, and not simply the primary outcome of the research study; (2) if researchers do not wish their participants to have access to feedback from the devices, then a simple, wrist-worn device that acts as a watch is preferable; (3) if feedback is allowed, then it should be made available to help participants remain engaged; this is likely to apply only to people without cognitive impairments; (4) battery life of 1 week should be considered as a necessary feature to enhance data capture; (5) researchers should consider providing additional information about the purpose of devices to participants to support their continued use.

## Introduction

### Background

The technological advancements of recent years are challenging the traditional methods of data capture within clinical trials. In particular, the use of wearable technology offers unprecedented access to a variety of accurate, objective health care data that can be captured remotely, thus providing real-time access to large amounts of patient data [1,2]. Wearable devices are considered more convenient for participants by enabling them to collect data themselves, potentially resulting in improved protocol compliance and retention [3].

Given the relatively recent development of wearable devices, research has primarily focused on evaluating their clinical validity [4]. However, in order for these devices to be successfully incorporated into clinical trials, not only must they reliably capture accurate data, but critically, participants must be willing to wear and engage with them over a sustained period. The International Organization for Standardization defines usability as the effectiveness, efficiency, and satisfaction with which specified users achieve specified goals in particular environments [5]. To evaluate these components, researchers need to understand the barriers and facilitators to the participant’s adherence with devices, to ensure that researchers do not inadvertently select clinically useful yet inappropriate devices, thus risking trial outcomes [6]. However, limited empirical evidence exists evaluating participant-centered usability of wearable devices within clinical trials [1], with wear-time and adherence rates used as proxy usability assessments. Furthermore, existing evaluations are limited by a focus on consumer-based products [7-10], short testing periods (ie, 24 hours or less) [11], the evaluation of a single wearable device only [12,13], and by the use of either qualitative or quantitative methods of data collection (but not both); thus, limiting the researchers full understanding of the participant’s experiences [14].

Given the increasing prevalence of chronic conditions, clinical trials that focus on cohorts of older adults will be a key focus of future research. Older adults often report of requiring assistance with technology [10,15,16], making it important to investigate the experiences of older adults with various wearable devices, particularly in those which are intended for medical and research environments, to understand which devices participants prefer wearing, and whether any barriers to their use exist. In particular, it is important that industry partners and research groups, who plan to run clinical trials, test a variety of devices in real-life remote monitoring situations that mirror the contexts and environments in which trials may take place.

### Objectives

Therefore, the primary aim of this study was to investigate the usability of a variety of wearable sensors in a real-world context by asking older adults to wear them in their home environment for a minimum of one week. Specifically, this was completed to establish the sensors’ utility and usability, beyond data quality, from the participant’s perspective and understand how these perceptions may affect their use in clinical trials.

## Methods

### Study Design and Participants

This was a six-week observational study that adopted mixed methods. No detailed inclusion or exclusion criteria existed; however, participants were required to be above 50 years of age, healthy, and fully independent in their daily lives. As this was an exploratory study, a power analysis was not undertaken. Eight participants from Dublin and the wider Wicklow and Kildare area, Ireland were recruited using purposive, convenience sampling through local flyers and existing connections between December 2017 and February 2018 to allow for comparisons of user experience, both between and within participants. Recruitment ceased once data saturation was reached in the qualitative analysis.

### Included Devices

Seven, small, noninvasive wearable sensor devices, designed to track activity and sleep data were selected: Actigraph GT9X Link (Actigraph LLC), Actibelt (Trium), Actiwatch Spectrum Plus (Philips), Biovotion Everion (Biovotion), Hexoskin (Carre Technology), Mc10 Biostamp_RC (MC10 Inc), and Wavelet (Wavelet Health; Table 1). These specific devices were selected by the industry partners of this study who wished to assess the usability of devices that may be used to track physical activity in future clinical trials. Devices were selected to compare the range of locations and level of user interaction that are available on the market for this purpose.

**Table 1:** Basic functional and usability information regarding the devices included within the study.

Device (manufacturer)	Tethered to	Intended use	User app required	User interface	Medical grade^a^	Battery life^b^	Memory capacity
Actigraph GT9X Link (Actigraph LLC) [17]	Wrist	Sleep, actigraphy, and energy expenditure	Yes (optional)	Watch screen	Yes	1 week	4 GB
Actibelt (Trium) [18]	Waist (flexbelt or leather belt	Actigraphy	No	None	No	3 months	1800 GB
Actiwatch Spectrum Plus (Philips) [19]	Wrist	Sleep and actigraphy	No	Watch screen	Yes	1 week	1 MB
Biovotion Everion (Biovotion) [20]	Upper arm	Heart rate, respiratory rate, actigraphy, skin temperature, heart rate variability, and oxygen saturation	Yes	None	Yes	24 hours	Server-based memory, 3 days of data capture on device
Hexoskin (Carre Technology) [21]	Torso	Heart rate and actigraphy	No	None	No	>24 hours	600 hours
Mc10 Biostamp_RC (Mc10 Inc) [22]	Upper thorax^c^	Heart rate and actigraphy	No	None	Yes	2-5 days	Server-based memory, 3 days of data capture on device
Wavelet (Wavelet Health) [23]	Wrist	Sleep and actigraphy	Yes	None	No	24-36 hours	Not reported

^a^Defined by manufacturers according to the Food and Drug Administration and European guidelines.

^b^As reported by the device manufacturer.

^c^In this study only, other attachment points exist.

### Study Procedure

At the entry point to the study, participants provided written informed consent, after which an opening interview was undertaken to establish their views on wearable technology in health and their previous experiences with wearable devices. Participants were then provided with a device and instructed to wear the device at all times (if possible, during their normal activities, except showering, for the duration of the week). Devices were worn for a full seven days each. The order of the devices was randomized to minimize bias. Depending on the device, participants were not required to interact with the device other than to charge them, if the device required. A week after the first testing session, participants returned their device and were provided with a new sensor. Participants were asked to complete three validated outcome measures (as described below); while semistructured interviews were completed at the end of each deployment week, so that feedback was provided specifically for each device independently. Upon completion of the study, participants completed a final semistructured interview, wherein they were asked about their overall perceptions of the included sensors within the study and which devices they preferred and why. Device deployment was randomized to limit the risk of bias.

### Data Collection and Outcome Measures

#### Quantitative Data Collection

Brief demographics of the participants were collected (ie, sex, age, height, weight, and any previous experience with sensors). In total, three questionnaires were given to each participant regarding each of the sensors.

- *The Systems Usability Scale (SUS)*: It measures the usability of a device/system/technology [24-26]. It consists of a 10-item questionnaire with five response options for respondents from *1: strongly disagree* to *5: strongly agree*, resulting in a potential minimum score of 0 and a maximum of 100.
- *Intrinsic Motivation Inventory (IMI)*: IMI is a multidimensional questionnaire intended to assess the participant’s experiences related to a target activity [27], in this case, wearing the wearable device. The instrument contains 22 items on a 7-point Likert scale, ranging from *1: not at all true* to *7: very true*. The measure assesses six subscales: *interest/enjoyment*, *perceived competence*, *effort/importance*, *pressure/tension*, *value/usefulness*, and *perceived choice*.
- *Acceptability questionnaire by Jacucci et al* [28]: Jacucci et al [28] aimed to assess users’ acceptance of wearable devices across dimensions including comfort, fear of technology, and privacy. Participants were asked to rate the extent to which they agreed or disagreed with each of the 26-item statements on a 6-point Likert scale ranging from *1: completely disagree* to *6: completely agree*, on 10 individual subsections.

#### Qualitative Data Collection

The aim of the qualitative phase was to explore the participant’s opinions of the devices and the factors they felt influenced their use of the same (interview guide provided in Multimedia Appendix 1). A female research physiotherapist (AK) with a PhD in behavior change (including two years of experience and training in qualitative research) and currently working in the area of digital health completed the semistructured interviews to extract more information from participants about certain aspects of the design or usability of the device. Interviews were completed in either participants’ homes or place of work, depending on their preference. Scratch notes were taken by AK during the interviews, which were also audio-recorded and transcribed verbatim by AK. As the sample was purposively gathered, some participants were known to the researcher and thus, a rapport was already established. Participants were aware of the purpose of the research through the participant information leaflet and consent form they signed before participating. Before completing the research, AK had pilot tested each device to ensure they were set up correctly; thus, she witnessed experiences of some of the potential barriers and facilitators to their use.

### Data Analysis

#### Quantitative Data Analysis

The SUS score was computed for each participant following standard scoring methodology [24]. Descriptive statistics were calculated to find out the median (IQR; min-max) result per device. To score the IMI, all negatively worded statements were inversely translated by subtracting the participant's score from eight. Following this, the average score for each of the six categories was calculated for each participant and group median (IQR; min-max) scores were calculated for each category for each device independently. A median result for the acceptability questionnaire was calculated per device, alongside a median result for each of its 10 subsections independently. In the absence of reference interpretations of the IMI and acceptability questionnaire, the midpoint of Likert scale was selected as the minimum level of acceptability of a device [29-32].

#### Qualitative Data Analysis

Deductive content analysis was undertaken for each of the transcribed texts using a realist approach, whereby the researcher assumed that the opinions of the participants reflected their true perceptions and should be taken as real [33]. A deductive content analysis was undertaken to categorize the participant’s responses based on previous knowledge [34]. Specifically, literature has suggested that perceived usefulness, comfort, and ease of use are critical factors of usability [10,35-37], thus, these were selected as the categories for which the content of the transcribed audio recordings would be assessed. In addition, because the research question focused on understanding whether participants would accept using these devices within a clinical trial, this was pragmatically selected as an additional category. Following the steps outlined in previous research [35], the researcher (AK) familiarized herself with the texts and then identified the content which corresponded with each of the preidentified categories [34,35]. Data saturation was deemed to have occurred when no additional learnings regarding the devices and their features were identified under the selected categories. This analysis was then discussed with another member of the research team (BR), who was experienced in qualitative research, to ensure accuracy in coding. Specific quotations, which were deemed to represent the most important aspects of participants’ experiences were selected for inclusion by AK and BR. Participant checking did not take place as part of this study, and transcripts were not provided to the participants.

#### Data Integration

A triangulation design was completed at the interpretation level of data analysis to provide a more complete picture of each device, to enhance the reliability of the study, and to support data saturation [38]. Specifically, a meta-matrix was created to facilitate comparisons of the results by presenting the quantitative data in tabular format alongside the summarized qualitative themes. For each sensor independently, all results were displayed on the same page, to determine whether there was convergence, partial convergence, discrepancy, or silence [39-42].

### Ethics Approval and Consent to Participate

This study received ethical approval from the University College Dublin Human Ethics Committee (ref: LS-17-92-Caulfield). All participants provided written informed consent.

## Results

### Demographic Information

Participant demographic information can be found in Table 2. Six participants reported feeling comfortable or very comfortable using technology. Three were wearing an activity tracker, while the remaining three had worn them in the past. The final two participants rated their technology comfort levels as medium, with no previous experiences of using wearable devices. All participants wore each of the seven devices, with the exception of Hexoskin. The reasons for which are outlined within the results. In addition, all participants reported wearing the devices at all times during the week, with the exception of Hexoskin. However, no formal assessment of adherence was completed.

**Table 2:** Participant demographic information.

Characteristic		Value
**Gender (n)**		
	Male	5
	Female	3
Age (years), mean (range)		62 (53-72)
**Level of education (n)**		
	Third level	3
	Secondary level	4
	Primary level	1
**Employment status (n)**		
	Retired	4
	Employed	4
**Experience with wearable devices (n)**		
	Yes (current or past)	6
	No	2

### Quantitative Results

#### System Usability Scale

The median score for all devices on the SUS was 57.5 (IQR 12.5; min-max=47.5-63.8) out of a possible score of 100. None of the tested devices were deemed to be *good* by participants, as all seven achieved scores of less than 68 (30). Actibelt achieved the highest median result of 63.8 (IQR 12.5; min-max=47.5-67.5), while Hexoskin achieved the lowest median result of 47.5; min-max=37.5- 57.5 (Table 3). The results for all of the devices fall between the 10th and the 30th percentile, meaning that all were considered below average [24].

#### Intrinsic Motivation Inventory

The median score for all devices on the IMI was 4.6 (1.0; 3.6-5.2) on the 7-point Likert scale. No device achieved very high results (Table 3). Hexoskin was the only device to score below the midpoint of Likert scale (3.6; 3.4-4.5), suggesting that participants would not be autonomously motivated to wear this device.

#### Acceptability Questionnaire

The median score for all devices on the acceptability questionnaire was 3.5 (0.5; 3.2-3.6). The highest median results were achieved by Biovotion, Actibelt, and Mc10 Biostamp_RC, with each achieving results of 3.6 on the 6-point Likert scale (Table 3).

**Table 3:** Participants’ self-reported usability of each device according to (1) Intrinsic Motivation Inventory, (2) System Usability Scale, and (3) Acceptability questionnaire.

Questionnaire domains		Actigraph, median (IQR); min-max	Actibelt, median (IQR); min-max	Actiwatch, median (IQR); min-max	Biovotion, median (IQR); min-max	Hexoskin^a^, median; min-max	Mc10, median (IQR); min-max	Wavelet, median (IQR); min-max	
**Intrinsic Motivation Inventory (n=22 questions; 7-point Likert scale)**									
	Median	4.3 (0.8); 3.9-5.4	4.1 (0.9); 3.3-5.1	4.7 (1.1); 3.0-5.4	5.2 (0.3); 4.0-5.5	3.6; 3.4-4.5	4.5 (1.1); 1.7-5.6		4.7 (0.8); 4.3-5.1
	Interest	3.5 (1.4); 2.3-5.3	3.4 (1.3); 2.8-5.0	4.5 (1.8); 1.5-5.5	6.0 (1.0); 2.5-7.0	3.5; 3.5-4.3	3.5 (1.0); 1.0-4.3		5.3 (0.6); 4.7-7.0
	Competence	6.7 (3.2); 2.7-7.0	6.2 (1.4); 5.3-7.0	6.3 (2.0); 4.7-7.0	6.5 (1.9); 3.4-7.0	4.3; 4.0-4.3	5.0 (1.7); 3.6-7.0		6.7 (0.8); 3.0-7.0
	Effort	3.3 (2.9); 2.0-5.8	3.8 (2.5); 2.3-5.5	3.5 (3.0); 2.5-6.3	3.9 (1.6); 2.0-5.8	3.5; 2.0-3.8	4.3 (2.8); 1.8-6.8		4.0 (1.3); 1.0-5.5
	Pressure	1.3 (2.0); 1.0-3.3	1.0 (0.3); 1.0-1.3	1.0 (2.0); 1.0-3.7	1.8 (1.7); 1.0-5.0	3.3; 2.0-3.7	3.0 (3.0); 1.0-4.0		2.0 (2.8); 1.0-4.0
	Choice	6.9 (0.9); 6.0-7.0	6.9 (1.4); 3.0-7.0	7.0 (1.0); 5.5-7.0	6.8 (1.5); 5.3-7.0	4.3; 4.0-7.0	6.8 (1.8); 1.5-7.0		4.0 (0.0); 3.3-4.0
	Usefulness	4.9 (2.5); 3.0-5.5	3.9 (2.1); 1.8-5.5	5.5 (3.0); 1.0-7.0	6.1 (1.7); 4.0-7.0	3.0; 1.8-3.3	5.0 (2.3); 1.0-6.8		6.8 (0.9); 5.0-7.0
**System Usability Scale (n=10 questions; 5-point Likert scale, score out of 100)**									
	Total score	60.0 (15.6); 50.0-67.5	63.8 (12.5); 47.5-67.5	57.5 (15.0); 50.0-65.0	56.6 (13.1); 45.0- 70.0	47.5; 37.5- 57.5	55.0 (12.5); 45.0-65.0		56.3 (9.4); 50.0-62.5
**Acceptability questionnaire (n=26 questions; 6-point Likert scale)**									
	Median score	3.6 (0.9); 2.8-5.2	3.4 (1.0); 2.8-4.7	3.2 (0.8); 3.0-4.0	3.6 (0.6); 3.0-4.8	3.2; 3.0-3.5	3.6 (0.4); 3.0-3.9		3.5 (0.4); 3.2-4.0
	Attitude	5.3 (1.6); 3.7-6.0	5.2 (1.1); 4.7-6.0	4.3 (1.0); 4.0-6.0	4.7 (1.8); 3.3-6.0	4.0; 3.3-4.7	4.3 (1.7); 4.0-5.7		4.3 (1.6); 3.7-6.0
	Anxiety	1.8 (2.5); 1.0-5.3	1.8 (2.6); 1.0-5.3	2.7 (1.7); 1.0-3.0	2.5 (2.9); 1.0-4.3	2.7; 2.3-2.7	3.0 (1.0); 2.3-3.7		2.3 (1.8); 1.0-5.0
	Facilitating conditions	2.5 (4.8); 1.0-6.0	2.5 (2.6); 1.0-4.0	1.5 (1.0); 1.0-3.0	2.5 (2.3); 1.0-3.5	5.5; 3.5-6.0	3.0 (4.5); 1.0-6.0		1.5 (1.0); 1.0-2.5
	Perceived usefulness	4.5 (2.7); 3.3-6.0	3.5 (2.3); 1.0-6.0	4.3 (2.3); 1.0-6.0	4.8 (1.5); 4.0-6.0	2.3; 1.3-3.0	3.3 (2.7); 1.0-6.0		5.2 (1.0); 4.0-6.0
	Perceived effort	3.8 (3.0); 3.0-6.0	3.8 (1.5); 3.5-5.0	3.5 (0.0); 3.5-4.0	4.5 (2.3); 3.0-6.0	5.0; 3.5-5.0	3.5 (1.0); 3.0-5.5		3.5 (0.5); 3.0-6.0
	Behavioral intentions	3.5 (1.4); 1.0-6.0	3.0 (1.2); 1.7-4.3	3.7 (1.0); 2.3-6.0	3.8 (0.8); 3.3-4.3	2.7; 2.7-3.0	3.0 (1.7); 2.7-4.7		3.8 (1.2); 3.0-4.3
	Psychological attachments	3.8 (2.1); 1.5-6.0	3.8 (2.4); 1.0-6.0	4.5 (3.0); 3.0-6.0	4.5 (1.8); 2.5-6.0	2.5; 1.5-3.5	3.0 (2.0); 1.0-6.0		4.0 (1.5); 1.5-5.0
	Privacy	2.5 (1.4); 1.0-5.0	3.0 (2.4); 1.0-6.0	2.5 (1.0); 1.0-5.0	3.3 (1.9); 1.0-4.5	2.5; 2.5-3.0	3.0 (3.0); 1.0-6.0		2.8 (1.6); 1.0-4.0
	Enjoyment	3.7 (1.5); 2.7-4.7	4.0 (1.1); 2.0-4.3	3.0 (1.0); 2.7-3.7	2.7 (1.1); 2.0-4.3	3.7; 3.0-4.3	3.7 (1.7); 1.7-5.0		2.7 (0.3); 1.0-3.0
	Comfort	3.3 (1.3); 2.0-5.3	4.3 (1.8); 2.3-4.3	4.0 (1.3); 2.7-4.3	4.0 (0.5); 3.7-4.7	3.0; 2.3-4.0	2.7 (1.3); 2.0-4.3		4.3 (0.7); 2.7-5.0

^a^n=3 participants. Hexoskin was removed from the study after receiving the feedback from the first three participants to use it. The burden they reported was considered too high to ask any remaining participants to use it. Therefore, no IQR exists.

### Qualitative Results

Interviews per device ranged from 10-21 min in length. Exit interviews at the end of the study ranged from 18 to 38 min in length. The findings for each device under the headings of *comfort of device*, *perceived usefulness of device*, *ease of use of device*, and *likelihood of wearing a device* are provided throughout the results with supporting quotations (participant numbers listed in parentheses).

#### Comfort of Devices

Participants believed that wrist-worn sensors were the most versatile and easy to use, and therefore, the most suitable for long-term use. In particular, Actiwatch and Wavelet stood out for their comfort. Wavelet, in particular, was remarked to be similar in design to *Fitbit*, resulting in its acceptability. However, the clasp method of closing the watch was not secure unless carefully completed, resulting in one participant losing a device. Actigraph was the only watch-based device that received negative feedback under the heading of comfort. The bulkiness of the device, perceived outdated design, and the frequency with which it *snagged* in participants’ clothes were the reasons for negative feedback.

Actibelt was perceived as surprisingly comfortable by all participants who expected it to be more cumbersome than it was. In contrast, Mc10 Biostamp_RC was notable for its lack of comfort. It was considered itchy. Participants noted that they were aware of Mc10 Biostamp_RC’s potential to fall off, while female participants were aware that the device was visible underneath certain clothing:

I just thought the most convenient and simplest one was the Wavelet. Well it was small, it was unobtrusive, it was a good design, it wasn’t as bulky as the Actigraph and it just looked like a normal kind of Fitbit.

101, male, age 64 years, employed

The ideal device is in a watch form because they are the easiest thing to wear, the ones that don’t interfere with day to day activities as much and they don’t interfere with what clothing you’re wearing, unless they’re very bulky.

401, female, age 56 years, employed

#### Ease of Use of Devices

The devices that required little to no interaction from participants were considered the easiest to use (ie, Actibelt, Actiwatch, and Actigraph GT9X Link). Although Mc10 Biostamp_RC did not require participants to engage with it, once it was on, participants were required to change the adhesive stickers every 1-3 days, resulting in the uncertainty and concern about the accuracy of their replacements; thus, the accuracy of the data provided by the device. In response, participants used the red marks on their skin left by the devices as guides to help them:

Oh I didn’t like the stamps [Mc10]...Well they were a bit fiddly to put on in the first place. They had the gel and it was hard to quite know the exact place to put them on, and then they can come off quite easily and then you have to put them back on...and then you have to take them on and off when you are having your shower, so they were almost completely impractical, certainly from a long-term point of view, you couldn’t do that for more than a couple of days.

101, male, age 64 years, employed

Wavelet and Biovotion provided participants with feedback through a mobile phone app, which was also the method required to monitor the battery level of the devices. For most participants this was not problematic, as the feedback provided by the device was interesting; therefore, engaging with the app was not a burden. However, the majority of participants agreed that long battery life was essential for long-term use of wearable devices, with a minimum of one week considered ideal. The need to charge a device daily was deemed unacceptable. Thus, this was a barrier to the sustained use of both Wavelet and Biovotion. One participant forgot to check the battery levels and as a result, missed the data collection of a number of days. In addition, Wavelet required users to select within the app, when they would go to sleep, resulting in an additional task, which was again, often forgotten:

Now perhaps if you have it for a long time you just purely get into the habit of doing it but it was very easy to forget because you know there’s I suppose, bed time you should get into procedures because I do, I remember to charge things to do stuff. If there was something that was on the device itself even if it was a little button that says sleep.

601, male, age 52 years, employed

The devices most difficult to use were also those that were the least favored. Specifically, Hexoskin was considered as an excessive burden on participants, as it required users to moisten the chest sensors within the vest frequently (every 15-20 min) to capture the heart and breathing rate data accurately. This was deemed impractical and disruptive to activities of daily living; therefore, a decision was made to cease the testing of the device, following the feedback from first three participants:

If you look at something like the vest [Hexoskin], which was very irritating that you had to keep wetting the sensors…I’d wear it for 24 hours but it’s not something that I would wear for a week and I certainly wouldn’t wear it for six weeks...No matter the feedback…because it’s just too limiting in your day to day activity…having to reach around under your breasts to find this piece of cloth that’s a sensor and then wet it is not something you can do easily in a public place.

401, female, age 56 years, employed

#### Perceived Usefulness of Devices

For the majority of participants, the best devices were those they felt they received the most feedback from (ie, Wavelet, Biovotion)*.* Indeed, participants seemed willing to compromise on *small annoyances* if they were personally getting something from the device. The devices with little to no feedback were not perceived as useful, with some participants appearing indifferent to the devices owing to this reason (ie, Actibelt, Actiwatch GT9X Link, and Mc10 Biostamp_RC). Nonetheless, participants were able to understand how these devices may still be valuable to others, including clinicians and researchers, and thus, were prepared to wear these devices *in the name of science*:

Well, because there was no feedback, it [Actiwatch] was pointless to me but in fairness to it was absolutely no trouble at all, you just forget it’s there, its design is better [than the Actigraph]. As you can see, I’m wearing it on a wrist with another watch and it just wasn’t an issue at all …it played no part in my life at all…first of all it’s just one piece, it’s got, even though it not much of a beveled edge, it’s got enough that things won’t snag on it as much. I do find it just sits better on the wrist the strap seems to be softer, more malleable.

601, male, age 52 years, employed

It’s there and it has no function [Actibelt]. There’s no feedback, there’s no information, there’s no feedback telling you what’s happening.

301, female, age 62 years, employed

Wavelet was reported to be the most useful device by participants who valued the simple graphs provided within the app (ie, sleep and heart rate). Actigraph GT9X Link was initially considered very basic, as the only information it provided was step count. Although, the participants did become accustomed to being able to easily check their step count throughout the day. Finally, even though Biovotion provided participants with innovative feedback (data were presented in an integrated spiral depicting a full day of information within a clock), the potential usefulness of future iterations of the device was greater than its current version. In particular, participants desired numerical data in addition to the spiral graph, to help them understand normal reference values. The suggestion by one participant that the device was *ahead of its time* is important, as it suggests that Biovotion is a promising product (dependent on future iterations) that may have a strong role to play in the monitoring of patient health:

I think it [Biovotion] was meant to measure things like your peripheral circulation or something, but again it gives you a number, it doesn’t tell you whether that means that your peripheral circulation is good, bad or indifferent…otherwise it’s just like a gimmick, it’s there you’ve got this little spiral that’s colourful, bit entertaining to look at…but you don’t get a chart to show what it was at various times during the day unless you just interpret what the spiral is showing,…all you get is real-time readings…it seems to be like the ultra-high definition televisions when they came out, they were fantastic, they looked wonderful but you couldn’t get ultra-high definition programs, so basically the televisions were head of its time. In a sense I think then maybe that this device is ahead of its time.

401, female, age 56 years, employed

#### Likelihood of Wearing a Device During a Trial

Participants agreed that the *purpose of use* is a key enabler for long-term compliance. These views were particularly noted by those not currently wearing an activity-tracking device. Although these participants explained that they did not personally feel the need to track their own activities, they suggested that they would not object to wearing a device for longer periods (ie, 8-12 weeks). For instance, in situations if they had to (ie, in the context of a clinical trial or by a clinician) and if the device was reasonably comfortable and easy to use. For most devices, participants reported that they would only wear them only if it was necessary, suggesting that their use of these devices would be born out of compliance rather than a specific, intrinsically motivated intention:

I would find it bothersome [having to wear the Mc10 within a trial]…I would be willing to do it you know because I think it’s good, but I was actually glad that today was the last day of these.

701, female, age 63 years, retired

I would do it for the sake of science, and for this, but I certainly wouldn’t, under no circumstances would I purchase it or use it kind of on an ongoing basis.

601, male, age 52 years, employed

### Integrated Results

Convergence was predominantly seen across each of the devices independently across the four headings: comfort, ease of use, usefulness, and likelihood of wearing the device. Specifically, an agreement could be observed between the qualitative and quantitative results overall; thus, providing support for each of the results. Table 4 provides a sample of this matrix, specifically for Actigraph GT9X Link. A full list of results for each individual sensor is available within Multimedia Appendix 2.

**Table 4:** Matrix of integrated qualitative and quantitative data for Actigraph GT9X Link (this device was used as an example).

Outcome of interest	Quantitative result, median (IQR); min-max	Qualitative result	Convergence; discrepancy; silence
Comfort	Midpoint of the Likert scale for perceived comfort (acceptability questionnaire): 3.3 (1.3); 2.0-5.3	Somewhat comfortable Unanimously agreed that the device was too big For some, along with excessive strap length, the device irritated them to the point of being uncomfortable Others felt that despite the size, the device was nonetheless comfortable	Convergence
Perceived usefulness	Midpoint for interest (IMI^a^): 3.5 (1.4); 2.3-5.3 Midpoint for usefulness (IMI): 4.9 (2.5); 3.0-5.5 Midpoint for effort/importance (IMI): 3.3 (2.9); 2.0-5.8 OK usability (SUS^b^): 60.0 (15.6); 50.0-67.5 High perceived usefulness (acceptability questionnaire): 4.5 (2.7); 3.3-6.0 Midpoint enjoyment (acceptability questionnaire): 3.7 (1.5); 2.7-4.7	Step count was both interesting and useful Further feedback was desired Device was considered *boring* due to its limited functionality Dual function as a watch appreciated	Convergence
Ease of use	High perceptions of competence (IMI): 6.7 (3.2); 2.7-7.0 Midpoint for perceived effort (acceptability questionnaire): 3.8 (3.0); 3.0-6.0 Midpoint for effort/importance (IMI): 3.3 (2.9); 2.0-5.8	Participants felt that the device was simple to use, as there was little to no interaction required with it Limited difficulties reported	Partial convergence
Likelihood of wearing a device	Low pressure to wear (IMI): 1.3 (2.0); 1.0-3.3 High perceived choice (IMI): 6.9 (0.9); 6.0-7.0 Midpoint behavioral intentions (acceptability questionnaire): 3.5 (1.4); 1.0-6.0 Midpoint psychological attachments (acceptability questionnaire): 3.8 (2.1); 1.5-6.0 Low facilitating conditions (acceptability questionnaire): 2.5 (4.8); 1.0-6.0	Participants were unclear whether this was a device suitable for long-term use The limited functionality is a plus for some and a barrier to others Almost everyone willing to wear it *for science* or if instructed by a health care professional Outside of a trial, the device was considered too bulky for long-term use Participants became used to it as the trial progressed; with many preferring it to other tested devices	Partial convergence

^a^IMI: Intrinsic Motivation Inventory.

^b^SUS: System Usability Scale.

## Discussion

### Principal Findings

This study aimed to investigate the usability of multiple wearables sensors within a real-world context by focusing on the human factors associated with their use in a group of older adults. This aim was achieved using mixed methods to determine participants’ likeliness to use and compliance with each device during a clinical trial; as judged through a week’s worth of constant wear. The results of this study further demonstrate the complexity involved in selecting a wearable device, as none of the tested sensors were considered optimal due to the influence of a variety of factors, including the feedback provided by the devices, their comfort, and their battery life.

### Comparison With Prior Work

A key strength of this study was the comparison of multiple devices within the same cohort of participants, thus offering an opportunity to accurately compare one device to another in the context of participants’ daily lives. The benefit of this multi-sensor approach, compared with other studies [11,43,44] was that within and between participant assessment of numerous devices, all with varying features and locations, our study allowed participants to note barriers that otherwise may not have been remarked without this easy and swift comparison. For example, Biovotion and Actibelt were noted for how little they interfered with activities of daily living, despite the initial expectation that they would be a burden. Furthermore, findings were strengthened by the use of mixed methods as the integrated findings typically converged; thus, demonstrating the robustness of the results. Although quantitative comparisons alone failed to provide a detailed understanding of why devices may differ, qualitative research does not always allow for generalizability. Integrating the two approaches provided a deeper understanding and comparison of what participants prioritized and favored within devices.

All devices in this study achieved SUS scores below average [45], suggesting they are only marginally usable. However, due to the small sample size in this study, these results should be interpreted with caution, as they cannot be generalizable to the wider population. In addition, the participants in this study were familiar with technology, which may limit direct comparisons with other research. Nonetheless, the quantitative results may provide some useful insights regarding the potential for these devices to be used in clinical trials. Specifically, low scores in the SUS are common, even among popular consumer devices including Fitbit [46]. A trade-off between comfort and functionality appears to exist, whereby participants are willing to accept a slightly less comfortable device, provided it serves a purpose that they value [47]. This is evidenced by participants consistently repeating that they would accept small annoyances for a device they perceived as beneficial. Indeed, it has been suggested that the “function of any wearable tool must outweigh any physical or social discomfort felt in wearing it, and less desirable devices may meet with higher standards for comfort and fit*.*” This finding echoes recent studies wherein participants were most likely to purchase and recommend devices based on their features, battery life, ease of use, and reliability [46,48-51]*.* Specifically, in relation to older adults, this study repeated the findings of previous research in that devices, which were deemed to be comfortable, fit seamlessly into daily routines, and demonstrated a clear perceived benefit to the participants were the devices that were favored [12,52]. Participants in this study consistently listed Wavelet and Biovotion as their preferred devices owing to the combination of useful feedback, comfort, and seamless interaction with their daily lives. However, the ability of participants to easily check the battery level of devices is a necessity, especially within a clinical trial wherein consistent data collection is paramount. Even though perceived usefulness and perceived ease of use are critical components for participants’ intention to use a wearable device [10,37], both Wavelet and Biovotion may be limited in the sense that their battery level needs to be regularly monitored by users.

Interestingly, participants have been shown to consistently select a favorite device, irrespective of the evidence they gather to refute this. This was mirrored in this study as participants overwhelmingly agreed that Actibelt was one of the most comfortable, least obtrusive devices, had the longest battery life, and yet consistently failed to list it as a favorite. The perceived importance of feedback is likely to be the sole reason for this discrepancy, therefore, highlighting one of the most important findings of this research: for participants to be motivated to wear a device, they must see a purpose for it. For example, Actibelt and Actiwatch were very comfortable to all participants; however, neither device provided feedback. As participants were not confident whether they understood what data were being collected, the devices were not considered useful by the participants. In contrast, Actigraph GT9X Link was cumbersome and bulky, yet its simple feedback made it a device that participants appreciated.

When the results of this study are combined with previous research [10,12,46], it is clear that participants in multiple cohorts, both healthy and clinical, are broadly accepting of wearable technology, and once they can see the use of a relatively comfortable devices, they will be willing to wear them. However, one important insight that needs to be considered by both researchers and device manufacturers alike; participants are often able to see the future capability of wearable technology beyond its current function and are often left disappointed by the realities of a device when compared with the potential (eg, the measurement of blood pressure with Biovotion). Thus, research investigating the usability of wearable devices is consistently strengthening the argument that user-centered design is critical for compliance, and that users must gain some sort of advantage from wearing these devices. For most users, this is gained through the provision of feedback. Although, it remains unclear as to what level of feedback is considered necessary by participants, especially within cohorts with cognitive impairments. Given that many medical devices are not routinely designed to provide feedback, the result of this is a clash between health and consumer attributes in cohorts that desire and can cognitively interpret it [51]. Indeed, a common research hypothesis is that wearable devices may alter clinical trial outcomes because of real-time metrics and the ability of users to self-monitor their behavior [53]. However, sustained and meaningful behavior change has yet to be consistently demonstrated through consumer-based wearables alone [36,37,46,53]. Therefore, it should be considered whether feedback is a tangible risk to clinical trial outcomes. If it is not a risk, the provision of feedback may be one of the most important variables to consider when selecting a device for users without a cognitive impairment, as its presence provides participants with a perceived value for the device, which may support enhanced compliance. In response to this, researchers need to consider whether they can select a device that provides participants with some form of feedback (eg, heart rate), while remaining blind to the primary outcome measure of the trial (eg, physical activity). This is in regard to the acknowledgement that the future device development needs to incorporate desired participant functions to enhance compliance.

### Limitations

The results of this study should be considered alongside its limitations. Firstly, the findings cannot be generalized to the wider population due to the small number of participants, specifically older adults, many of whom were comfortable with technology. Thus, the findings of this study cannot be widely generalizable. However, as technology becomes more pervasive, older participants will become accustomed to its use, and thus, understanding the experiences of those who are comfortable with technology is nonetheless useful. Indeed almost 80% of older adults in one study reported using some form of technology in their lives [13]; however, it must be acknowledged that the experiences of people in their mid-60s cannot be compared with those in their 70s or above [13]. Additionally, although eight participants is a small number, participants acted as their own controls by comparing the use of multiple devices, thus, providing valuable within-study comparisons. Furthermore, the clinical utility and accuracy of these devices was not evaluated as part of this study. However, since this study commenced, some manufacturers have, or are about to release new versions of these devices on the market (eg, Actigraph). In addition, no formal measure of wear-time was collected within this study. Therefore, the results rely on participants’ self-report of whether they used the device or not. However, given that the focus of this study was on the usability of the device, compliance was not considered an important quantitative variable. For instance, in the case of Hexoskin, participants made it clear that they would not comply, and did not continue to wear the device due to its lack of usability. Given the aim of this study, this qualitative finding was more valuable than a quantitative measure of compliance as they highlighted the reasons why compliance was poor rather than simply whether it was or not. Finally, the result for Mc10 Biostamp_RC are likely to have been negatively influenced by the placement of the sensors on the pectoral muscles of participants, while Hexoskin is not intended for long-term monitoring. Future research should deploy the Mc10 Biostamp_RC device on alternative locations to determine whether the findings seen here are replicated. Since completing this study, the Biostamp_RC has been discontinued by Mc10 and has been replaced by Biostamp nPoint. Despite these limitations, the recommendations within this study may be of practical support for researchers considering which device to use within their trials.

### Conclusions

By using mixed methods and testing each device for a week, this study gained a robust understanding of the complexities of selecting a device for use within a clinical trial. The results indicate that no single sensor was considered optimal by participants due to a variety of factors, including the feedback provided by the device, its comfort, and battery life. Participants favored devices that they perceived they gained value from and were willing to overlook annoyances to receive feedback. Based on these results, the following context-specific recommendations can be made:

1. Researchers should consider their device selection in relation to both individual and environmental factors and not simply the primary outcome of the research study.
2. If researchers do not wish their participants to have access to the feedback from the devices, then a simple, wrist-worn device that acts as a watch is preferable.
3. If feedback is allowed, then it should be made available to help keep participants engaged. This is likely to apply only to people without cognitive impairments.
4. Battery life of 1 week should be considered as a necessary feature to enhance data capture.
5. Researchers should consider providing additional information about the purpose of devices to participants to support their continued use.

## Appendix

Multimedia Appendix 1 Interview guide.

Multimedia Appendix 2 Full list of tables of triangulated data per device.

## Abbreviations

IMI: Intrinsic Motivation Inventory
SUS: System Usability Scale
