# Supplementary material for: Comparing the Usability and Acceptability of Wearable Sensors Among Older Irish Adults in a Real-World Context: Observational Study
Source: JMIR Mhealth Uhealth. 2020 Apr 20;8(4):e15704. doi: 10.2196/15704 (PMC7199137; doi:10.2196/15704)
Supplement: Multimedia Appendix 1 [file mhealth_v8i4e15704_app1.docx]

## **Interview Guide**

### **Entry interview**

Open with background questions regarding:

- Age
- Education
- Employment info
- Physical Activity Level
- Health Status

Technology

- How familiar are you with ‘technology’? (Phones, Tablets, Actigraphy etc)
- Do you currently/previously have experience with electronic journals/ wearables?
  - Prompts include
    - Why / Why not?
    - Which ones?
    - How have you liked/disliked them?
    - If no experience, or if no longer using devices probe the following:
      - *Is there anything that you think would make you use them?*
      - *Is there anything that would make you want to use them or feel like you need to use them?*
      - *Do they feel like they need to use them? What would make you feel like you need to use them?*
- Do you keep track of activity/sleep/pain in written form(other means)?
- How confident are you with tech?
  - Prompts include
    - Do they think they need to improve their skills with tech? Would this help them or motivate them to use it?
- How do you feel about having two sensors monitoring you at once?
- What is your opinion on GP/Physio/Doc/Nurse monitoring activity and remotely access data through the use of wearables?
  - Prompts include:
    - And, what if device could notify Practitioner when decline happens?
    - Is it useful for you to monitor your activity?
    - Why?
    - Do they need more information about why or how they work?
    - Are there any perceived barriers to using them?
- What is your opinion on the use of these device as monitoring devices?
  - Is it useful for you to monitor your activity?
  - Why?
  - Do they need more information about why or how they work?
  - Are there any perceived barriers to using them?
  - How do you feel your access to or awareness of these devices is currently?
  - If more would it be likely to make them use them?
    - Money/time/support from others etc.

General Health

- In General, how is your health?
  - - Have you a routine/habits to help with pain/stress/fatigue/diet?
- How do you think wearing an activity monitor will impact your daily routine?
  - - How and why?

### **Transition interview per device**

Usability

- What were your first impressions of the device?
- How did you find using the sensor this week?
- What Did you Like/Dislike ?
  - What and why
- Had you any issues/problems?
  - What and why
  - How did it influence their use?
- How easy was the device to use, this week?
  - Probe more
- Did wearing the device have any affect on your activity/sleep?
  - If so what and why? Good or bad?
- Did you have any reason to remove either the devices?
  - - Why / Why not?
- Were you comfortable wearing the devices in public?
  - - Did people ask about either of them?
- Did you find any barriers to using the device?
- How was the reporting platform on the app?
  - - Easy to use?
    - Enjoyable/Informing?
- If you had to wear this device as a part of a trial what would be the barriers to participating in this trial for you?
- Length of time necessary to wear
- Interaction level required
- Desired device feedback etc?
- What would you change about the devices, and its app (if appropriate)?
- Would you recommend the devices?
  - Why/ why not?
  - What would need to change before you would recommend it?
  - Would they use it/purchase it themselves if they had the choice?
    - Why?
    - If no, what would need to change?
      - Do they feel they understood it/had the skills to use it? Do they feel confident that they knew how to use it?
      - Would they be motivated?
      - Do they like they would want to or need to use it?
      - Money/time/others using it?
      - Would triggers or prompts help them use it?

### **Exit interview**

- Which devices did you prefer?
  - - Why?
- Were the smartphone apps easy to use?
  - Which in particular? Why or why not?
- Were you comfortable/uncomfortable being remotely monitored through the study?
- Did wearing devices cause change in your usual habits? Sleep/activity etc?
  - If so how
  - If not, why? What would need to change for them to develop the habit of using devices or engaging with them?
    - E.G. what are their opinions on their knowledge of the sensors and how to use them is?
    - How do they feel their skills with using the sensors was?
    - Is there any feelings or obstacles they feel they need to overcome to help use them?
    - Are there any barriers they can think of to forming a habit of using them?
      - Time, money, support from others?
    - Anything else?
  - How was your desire to use them/engage with them during the study
    - Why poor or good?
    - What were your impressions of what you would get out of using them?
      - Did they feel it would be beneficial or poor?
    - Anything else
    - Probe these answers more depending on responses.
    - Would they be likely to use any of these volitionally, or purchase monitoring devices in the future based on their experiences here?
  - Do they feel like they needed to use them/engage with them during the study
    - Why or why not?
    - Anything else
    - Probe these answers more depending on responses
- What made you take off devices?
  - Which in particular?
  - Were there any that they were very comfortable wearing?
- Was it uncomfortable wearing the device in public?
  - - Did it become a topic of conversation?
