# Supplementary material for: Comparing the Usability and Acceptability of Wearable Sensors Among Older Irish Adults in a Real-World Context: Observational Study
Source: JMIR Mhealth Uhealth. 2020 Apr 20;8(4):e15704. doi: 10.2196/15704 (PMC7199137; doi:10.2196/15704)
Supplement: Multimedia Appendix 2 [file mhealth_v8i4e15704_app2.docx]

**Supplemental file 2 – Full list of integrated results from each of the included devices.**

1. **Matrix of integrated qualitative and quantitative results for the Actigraph**

| **Outcome of interest** | **Quantitative results** | **Qualitative results** | **Convergence; Discrepancy; Silence** |
| --- | --- | --- | --- |
| **Comfort** | Mid- point of the Likert scale for perceived comfort (Acceptability questionnaire): 3.3 (1.3); 2.0-5.3 | Somewhat comfortable. | Convergence |
|  |  | Unanimously agreed that the device was too big. |  |
|  |  | For some this, along with excessive strap length, irritated them to the point of being uncomfortable. |  |
|  |  | Others felt that despite the size, the device was nonetheless comfortable. |  |
|  |  |  |  |

| **Ease of use** | High perceptions of competence (IMI): 6.7 (3.2); 2.7-7.0 | Participants felt that the device was simple to use as there was little to no interaction required with it. | Partial convergence |
| --- | --- | --- | --- |
|  | Mid-point for perceived effort (Acceptability questionnaire): 3.8 (3.0); 3.0-6.0 | Limited difficulties reported |  |
|  | Mid-point for effort/importance (IMI): 3.3 (2.9); 2.0-5.8 |  |  |
|  |  |  |  |
| **Usefulness** | Mid-point for interest (IMI): 3.5 (1.4); 2.3-5.3 | Step count was both interesting and useful. | Convergence |
|  | Mid-point for usefulness (IMI): 4.9 (2.5); 3.0-5.5 | Further feedback was desired. |  |
|  | Mid-point for effort/importance (IMI): 3.3 (2.9); 2.0-5.8 | Device considered ‘boring’ due to its limited functionality. |  |
|  | ‘OK’ usability (SUS): 60.0 (15.6); 50.0-67.5 | Dual function as a watch appreciated. |  |
|  | High perceived usefulness (Acceptability questionnaire): 4.5 (2.7); 3.3-6.0 |  |  |
|  | Mid-point enjoyment (Acceptability questionnaire): 3.7 (1.5); 2.7-4.7 |  |  |
|  |  |  |  |
| **Likelihood/Intention to wear** | Low pressure to wear (IMI): 1.3 (2.0); 1.0-3.3 | Participants were unclear whether this was a device suitable for long-term use. | Partial convergence |
|  | High perceived choice (IMI): 6.9 (0.9); 6.0-7.0 | The limited functionality is a plus for some, and a barrier to others. |  |
|  | Mid-point behavioural intentions (Acceptability questionnaire): 3.5 (1.4); 1.0-6.0 | Almost all willing to wear it ‘for science’ or if instructed to by a healthcare professional. |  |
|  | Mid-point psychological attachments (Acceptability questionnaire): 3.8 (2.1); 1.5-6.0 | Outside of a trial the device was considered too bulky for long-term use. |  |
|  | Low facilitating conditions (Acceptability questionnaire): 2.5 (4.8); 1.0-6.0 | Nonetheless, participants became used to it as the trial progressed, with many preferring it to other tested devices. |  |

1. **Matrix of integrated qualitative and quantitative results for the Actibelt**

| **Outcome of interest** | **Quantitative results** | **Qualitative results** | **Convergence; Discrepancy; Silence** |
| --- | --- | --- | --- |
| **Comfort** | Above mid- point of the Likert scale for perceived comfort (Acceptability questionnaire): 4.3 (1.8); 2.3-4.3 | Very comfortable. | Convergence |
|  |  | Surprisingly comfortable to wear overall |  |
|  |  | Tendency to travel up the torso |  |
|  |  | Heavy and ‘bulky’ belt buckle |  |
|  |  |  |  |
| **Ease of use** | High perceptions of competence (IMI): 6.2 (1.4); 5.3-7.0 | Participants felt that the device was simple to use as there was no interaction required with it. | Partial convergence |
|  | Mid-point for perceived effort (Acceptability questionnaire): 3.8 (1.5); 3.5-5.0 | No difficulties reported |  |
|  | Just at mid-point for effort/importance (IMI): 3.8 (2.5); 1.8-5.5 |  |  |
|  |  |  |  |
| **Usefulness** | Below mid-point for interest (IMI): 3.4 (1.3); 2.8-5.0 | Lack of feedback renders the device useless for participants in a normal, day-to-day setting | Discrepancy |
|  | Just at mid-point for usefulness (IMI): 3.9 (2.1); 1.8-5.5 | Participants unsure as to what the device was measuring or doing. |  |
|  | Just at mid-point for effort/importance (IMI): 3.8 (2.5); 1.8-5.5 |  |  |
|  | ‘OK’/ below average usability (SUS): 63.8 (12.5); 47.5-67.5 |  |  |
|  | Mid-point perceived effort (Acceptability questionnaire): 3.8 (1.5); 3.5-5.0 |  |  |
|  | High enjoyment (Acceptability questionnaire): 4.0 (1.8); 2.3-4.3 |  |  |
|  | Mid-point perceived usefulness (Acceptability questionnaire): 3.5 (2.3); 1.0-6.0 |  |  |
|  |  |  |  |
| **Likelihood/Intention to wear** | Low pressure to wear (IMI): 1.0 (0.3); 1.0-1.3 | Participants did not feel that this was a device suitable for long-term use, due to its lack of feedback. | Convergence |
|  | Mid-point behavioural intentions (Acceptability questionnaire): 3.7 (1.0); 2.3-6.0 | Majority willing to wear it within a trial even with no feedback |  |
|  | Low facilitating conditions (Acceptability questionnaire): 2.5 (2.6); 1.0-4.0 | More willing to use this without feedback compared to other tested devices. |  |
|  |  |  |  |

1. **Matrix of integrated qualitative and quantitative results for the Actiwatch**

| **Outcome of interest** | **Quantitative results** | **Qualitative results** | **Convergence; Discrepancy; Silence** |
| --- | --- | --- | --- |
| **Comfort** | Mid- point of the Likert scale for perceived comfort (Acceptability questionnaire): 4.0 (1.3); 2.7-4.3 | Very comfortable. | Convergence |
|  |  | Smaller than the Actigraph |  |
|  |  | Slightly bevelled edges reduced the potential for snagging in clothes |  |
|  |  |  |  |
| **Ease of use** | High perceptions of competence (IMI): 6.3 (2.0); 4.7-7.0 | Participants felt that the device was simple to use as there was no interaction required with it. | Partial convergence |
|  | Mid-point for perceived effort (Acceptability questionnaire): 3.5 (0.0); 3.5-4.0 | No difficulties reported |  |
|  | Mid-point for effort/importance (IMI): 3.5 (3.0); 2.5-6.3 |  |  |
|  |  |  |  |
| **Usefulness** | Mid-point for interest (IMI): 4.5 (1.8); 1.5-5.5 | Lack of feedback was disappointing and renders the device useless for participants | Partial convergence |
|  | Mid-point for usefulness (IMI): 4.9 (2.5); 3.0-5.5 | Participants understood the potential use and benefits for healthcare providers to remotely monitor them or gather helpful feedback from the device |  |
|  | Mid-point for effort/importance (IMI): 3.5 (3.0); 2.5-6.3 | Participants unsure as to what the device was measuring or doing. |  |
|  | ‘OK’/ below average usability (SUS): 57.5 (15.0); 50.0-65.0 |  |  |
|  | High perceived usefulness (Acceptability questionnaire): 4.3 (2.3); 1.0-6.0 |  |  |
|  | Mid-point enjoyment (Acceptability questionnaire): 3.0 (1.0); 2.7-437 |  |  |
|  |  |  |  |
| **Likelihood/Intention to wear** | Low pressure to wear (IMI): 1.0 (2.0); 1.0-3.7 | Participants did not feel that this was a device suitable for long-term use, due to its lack of feedback. | Partial convergence |
|  | High perceived choice (IMI): 7.0 (1.0); 5.5-7.0 | All willing to wear it within a trial or if instructed to by a healthcare professional. |  |
|  | Mid-point behavioural intentions (Acceptability questionnaire): 3.7 (1.0); 2.3-6.0 | For it to be used by participants daily, they would require some form of feedback |  |
|  | Low facilitating conditions (Acceptability questionnaire): 1.5 (1.0); 1.0-3.0 | Most participants were more willing to use a less comfortable device (i.e. the Actigraph) if it provided them with feedback. |  |
|  |  |  |  |

1. **Matrix of integrated qualitative and quantitative results for the Biovotion**

| **Outcome of interest** | **Quantitative results** | **Qualitative results** | **Convergence; Discrepancy; Silence** |
| --- | --- | --- | --- |
| **Comfort** | High reported comfort (Acceptability questionnaire): 4.0 (0.5); 3.7-4.7 | Very comfortable. | Convergence |
|  |  | Many did not realise it was there. |  |
|  |  | Minor issues relating to the device sticking to skin. |  |
|  |  |  |  |
| **Ease of use** | High perceptions of competence (IMI): 6.5 (1.9); 3.4-7.0 | Daily charging is a high burden for participants. | Partial convergence |
|  | High perceived effort (Acceptability questionnaire): 4.5 (2.3); 3.0-6.0 | Limited difficulties reported in terms of using the app or putting on the device |  |
|  | Mid-point for effort/importance (IMI): 3.9 (1.6); 2.0-5.0 | However, the feedback is difficult to interpret without reference values or information on variables |  |
|  | Below average SUS: 56.6 (13.1); 45.0-70.0 |  |  |
|  |  |  |  |
| **Usefulness** | High result for interest (IMI): 6.0 (1.0); 2.5-7.0 | Representation of the data was interesting. | Partial convergence |
|  | High result for usefulness (IMI): 6.1 (1.7); 4.0-7.0 | Device considered a gimmick or ahead of its time |  |
|  | Mid-point for effort/importance (IMI): 3.9 (1.6); 2.0-5.0 | Potential of the device greater than its current function |  |
|  | ‘Below average’ SUS: 56.6 (13.1); 45.0-70.0 | The ability to see patterns in health over a prolonged period was useful, but requires numerical figures to accompany it |  |
|  | High perceived usefulness (Acceptability questionnaire): 4.5 (2.7); 3.3-6.0 | If blood pulse wave is shown to be an accurate predictor of cardiovascular risk, this device holds huge potential |  |
|  | Low result for enjoyment (Acceptability questionnaire): 2.7 (1.1); 2.0-4.3 |  |  |
|  |  |  |  |
| **Likelihood/Intention to wear** | Low pressure to wear (IMI): 1.8 (1.7); 1.0-5.0 | Participants were unclear whether this was a device currently suitable for long-term use. | Partial convergence |
|  | High interest (IMI): 6.0 (1.0); 2.5-7.0 | Positive reaction regarding its use within a clinical trial |  |
|  | Mid-point behavioural intentions (Acceptability questionnaire): 3.8 (0.8); 3.3-4.3 | Improved feedback would increase adherence |  |
|  | Low facilitating conditions (Acceptability questionnaire): 2.5 (2.3); 1.0-3.5 |  |  |
|  |  |  |  |

| **5) Integrated qualitative and quantitative data matrix for the Hexoskin** | | | |
| --- | --- | --- | --- |
| **Outcome of interest** | **Quantitative results** | **Qualitative results** | **Convergence; Discrepancy; Silence** |
| **Comfort** | Mid-point of the Likert scale for perceived comfort (Acceptability questionnaire): 3.0; 2.3-4.0 | Comfortable during the day | Convergence |
|  |  | The placement of the device in the pocket is uncomfortable at night |  |
|  |  | The vest is somewhat tight to put on and better suited to sports people |  |
|  |  |  |  |
| **Ease of use** | Mid-point perceptions of competence (IMI): 4.3; 4.0-4.3 | Not practical or easy to use due to the need to frequently wet the sensors | Convergence |
|  | High perceived effort (Acceptability questionnaire): 5.0 (3.5-5.0) | The need to frequently charge the battery was a burden |  |
|  | Mid-point for effort/importance (IMI): 3.5 (2.0-3.8) | Wetting the sensors in public for females is not easy |  |
|  | Poor SUS: 47.5; 37.5-57.5 | The app drains phone battery |  |
|  |  | Impractical to wear daily if you also want to exercise unless you purchase multiple vests per person. |  |
|  |  |  |  |
| **Usefulness** | Below mid-point for interest (IMI): 3.5; 3.5-4.3 | Feedback may be useful but cannot be trusted due to the need to wet the sensors so often | Convergence |
|  | Below mid-point for usefulness (IMI): 5.0; 1.8-3.3 | Participants would not wear this daily |  |
|  | Poor SUS: 47.5; 37.5-57.5 | The device is too disruptive to their daily lives and their inability to trust the data would result in a lack of compliance |  |
|  | Below mid-point perceived usefulness (Acceptability questionnaire): 2.3; 1.3-3.0 |  |  |
|  | Mid-point enjoyment (Acceptability): 3.7); 3.0-4.3 |  |  |
|  |  |  |  |
| **Likelihood/Intention to wear** | Below mid-point for interest (IMI): 3.5; 3.5-4.3 |  | Convergence |
|  | High facilitating conditions (Acceptability questionnaire): 5.5 (3.5- 6.0) |  |  |
|  | Low behavioural intentions (Acceptability questionnaire): 2.7 (2.7-3.0) |  |  |
|  |  |  |  |

| **6) Integrated qualitative and quantitative data matrix for the Mc10_Biostamp** | | | |
| --- | --- | --- | --- |
| **Outcome of interest** | **Quantitative results** | **Qualitative results** | **Convergence; Discrepancy; Silence** |
| **Comfort** | Below mid-point of the Likert scale for perceived comfort (Acceptability questionnaire): 2.7 (1.3); 2.0-4.3 | Not very comfortable | Convergence |
|  |  | Itching around the edges of the device was common, though mild |  |
|  |  | Redness was visible upon removal of the device |  |
|  |  | Some feared losing the device creating a low level of persistent anxiety |  |
|  |  |  |  |
| **Ease of use** | High perceptions of competence (IMI): 5.0 (1.7); 3.6-7.0 | Participants felt that the device was simple to use as there was little to no interaction required with it. | Convergence |
|  | Mid-point for perceived effort (Acceptability questionnaire): 3.5 (1.0); 3.0-5.5 | The replacement of the adhesives was considered to be a burden |  |
|  | Mid-point for effort/importance (IMI): 4.3 (2.8); 1.8-6.8 | Uncertainty regarding the placement of the devices created some concern |  |
|  | Below average SUS: 55.0 (12.5); 45.0-65.0 | The placement of the device on the chest resulted in participants having to actively choose appropriate clothing |  |
|  |  |  |  |
| **Usefulness** | Below mid-point for interest (IMI): 3.5 (1.0); 1.0-4.3 | Without feedback the device was not considered useful | Partial convergence |
|  | Above mid-point for usefulness (IMI): 5.0 (2.3); 1.0-6.8 | No functions to discuss resulting in some participants reporting indifference towards the device. |  |
|  | Below average SUS: 55.0 (12.5); 45.0-65.0 | Some perceived value for healthcare practitioners who may be able to use the data |  |
|  | Mid-point perceived usefulness (Acceptability questionnairr): 3.3 (2.7); 1.0-6.0 |  |  |
|  | Mid-point enjoyment (Acceptability questionnaire): 3.7 (1.7); 1.7-5.0 |  |  |
|  |  |  |  |
| **Likelihood/Intention to wear** | Below mid-point for interest (IMI): 3.5 (1.0); 1.0-4.3 | Participants would not wish to use this long-term, particularly without feedback | Partial convergence |
|  | High perceived choice (IMI): 6.8 (1.8); 1.5-7.0 | Some reported being relieved that this only lasted a week |  |
|  | Mid-point behavioural intentions (Acceptability questionnaire): 3.0 (1.7); 2.7-4.7 | Participants were less supportive of its use during a trial compared to other devices |  |
|  | Mid-point psychological attachments (Acceptability questionnaire): 3.0 (2.0); 1.0-6.0 | The placement of the devices on the chest is likely to have negatively influenced their perceptions |  |
|  | Low facilitating conditions (Acceptability questionnaire): 3.0 (4.5); 1.0-6.0 |  |  |

| **7) Integrated qualitative and quantitative data matrix for the Wavelet** | | | |
| --- | --- | --- | --- |
| **Outcome of interest** | **Quantitative results** | **Qualitative results** | **Convergence; Discrepancy; Silence** |
| **Comfort** | Above mid-point of the Likert scale for perceived comfort (Acceptability questionnaire): 4.3 (0.7); 2.7-5.0 | Very comfortable | Convergence |
|  |  | Good design and inconspicuous |  |
|  |  | Somewhat difficult to close |  |
|  |  | Fear of losing the device in some participants due to the mechanism of closing the strap |  |
|  |  | One device was lost during testing because of the strap |  |
|  |  |  |  |
| **Ease of use** | High perceptions of competence (IMI): 6.7 (0.8); 3.0-7.0 | The device was simple to use and the accompanying app simple to navigate | Convergence |
|  | Mid-point for perceived effort (Acceptability questionnaire): 3.5 (0.5); 3.0-6.0 | The feedback was easy to understand and the graphs are user friendly |  |
|  | Mid-point for effort/importance (IMI): 4.0 (1.3); 1.0-5.5 | The need to frequently charge the device was frustrating |  |
|  | Below average SUS: 56.3 (9.4); 50.0-62.5 | The charging dock was ‘fiddly’ to use |  |
|  |  | Having to check the battery through the app was problematic as you could forget about it, while patience was needed to allow for automatic syncing |  |
|  |  |  |  |
| **Usefulness** | Below mid-point for interest (IMI): 5.3 (0.6); 4.7-7.0 | The feedback was very interesting to participants | Convergence |
|  | High usefulness (IMI): 6.8 (0.9); 5.0-7.0 | The need to actively record sleep was problematic, as participants may easily forget and again had to wait for the device to sync. |  |
|  | Below average SUS: 56.3 (9.4); 50.0-62.5 | Some participants could see the benefit to using the device during everyday life. |  |
|  | High perceived usefulness (Acceptability questionnaire): 5.2 (1.0); 4.0-6.0 |  |  |
|  | Low enjoyment (Acceptability questionnaire): 2.7 (0.3); 1.0-3.0 |  |  |
|  |  |  |  |
| **Likelihood/Intention to wear** | Below mid-point for interest (IMI): 5.3 (0.6); 4.7-7.0 | Participants would be willing to wear the device in a trial. | Convergence |
|  | High psychological attachments (Acceptability questionnaire): 4.0 (1.5); 1.5-5.0 | Participants would be happy to wear the device even if blind to the feedback. |  |
|  | Low facilitating conditions (Acceptability questionnaire): 1.5 (1.6); 1.0-2.5 | Compared to other devices, the benefits of the Wavelet to their daily lives was seen or considered. |  |
|  | Mid-point behavioural intentions: 3.8 (1.2); 3.0-4.5 | Some participants would be willing to purchase it now, others if they had a health-related issue. |  |
